# Self‐management of first trimester medical termination of pregnancy: a qualitative study of women's experiences

**DOI:** 10.1111/1471-0528.14690

**Published:** 2017-06-14

**Authors:** C Purcell, S Cameron, J Lawton, A Glasier, J Harden

**Affiliations:** ^1^ MRC/CSO Social and Public Health Sciences Unit University of Glasgow Glasgow UK; ^2^ Chalmers Centre for Sexual and Reproductive Health (NHS Lothian) Edinburgh UK; ^3^ Usher Institute of Population Health Sciences and Informatics University of Edinburgh Edinburgh UK; ^4^ Obstetrics and Gynaecology Queen's Medical Research Institute University of Edinburgh Edinburgh UK

**Keywords:** First trimester medical/medication abortion, home self‐management, qualitative research, termination of pregnancy, women's experiences

## Abstract

**Objective:**

To explore the experiences of women in Scotland who return home to complete medical termination of pregnancy (TOP) ≤63 days of gestation, after being administered with mifepristone and misoprostol at an NHS TOP clinic.

**Design:**

Qualitative interview study.

**Setting:**

One National Health Service health board (administrative) area in Scotland.

**Population or Sample:**

Women in Scotland who had undergone medical TOP ≤63 days, and self‐managed passing the pregnancy at home; recruited from three clinics in one NHS health board area between January and July 2014.

**Methods:**

In‐depth, semi‐structured interviews with 44 women in Scotland who had recently undergone TOP ≤63 days of gestation, and who returned home to pass the pregnancy. Data were analysed thematically using an approach informed by the Framework method.

**Main outcome measures:**

Women's experiences of self‐management of TOP ≤63 days of gestation.

**Results:**

Key themes emerging from the analysis related to self‐administration of misoprostol in clinic; reasons for choosing home self‐management; facilitation of self‐management and expectation‐setting; experiences of getting home; self‐managing and monitoring treatment progress; support for self‐management (in person and remotely); and pregnancy self‐testing to confirm completion.

**Conclusions:**

Participants primarily found self‐administration of misoprostol and home self‐management to be acceptable and/or preferable, particularly where this was experienced as a decision made jointly with health professionals. The way in which home self‐management is presented to women at clinic requires ongoing attention. Women could benefit from the option of home administration of misoprostol.

**Tweetable abstract:**

Women undergoing medical TOP 63 days found home self‐management to be acceptable and/or preferable.

## Tweetable abstract

Women undergoing medical TOP 63 days found home self‐management to be acceptable and/or preferable.

## Introduction

Recent World Health Organization guidance includes the option of home self‐management for first trimester medical termination of pregnancy (TOP).^1^ However, the current interpretation of British legislation^1^– the 1967 Abortion Act which governs provision in Scotland, Wales and England – is that both TOP medications (mifepristone and misoprostol) must be administered on National Health Service (NHS) premises. With the development of safe, reliable treatment regimens for TOP ≤63 days of gestation has come the option for women in Britain to return home to self‐manage passing the pregnancy, *after* misoprostol has been administered at an outpatient clinic.

Research suggests that women self‐managing first trimester TOP have varied experiences^2^, ^3^, ^4^ but are no more likely to re‐attend with complications than those treated in hospital.^5^ Although a growing body of literature suggests that self‐management is acceptable to women,^2^, ^6^, ^7^, ^8^, ^9^, ^10^, ^11^, ^12^, ^13^, ^14^, ^15^, ^16^, ^17^ there is a need to monitor and understand women's needs around this model of provision, to facilitate provision of the best possible support. Our research contributes to this understanding, by examining details of women's experiences of self‐management ≤63 days, of which to date, relatively little is known.

In Britain, home self‐management is becoming increasingly established practice, and is now the default option for eligible women in many NHS and independent clinics.^2^ ^14^, ^15^ Women are also commonly given the option vaginally to self‐administer misoprostol at the clinic. This paper draws on data from one Scottish NHS health board area, where women are typically treated over two clinic visits,^18^, ^19^ and in which around 77% of TOP ≤63 days (55% of all terminations) are completed at home.^5^ The first visit involves counselling and administering mifepristone; and the second, provision of misoprostol and post‐TOP contraception.^18^ At the time of fieldwork, clinic protocol specified that women should be given a choice between home self‐management and hospital care.^3^

The objective of the wider study was to evaluate provision of TOP ≤63 days from the perspectives of women and health professionals.^18^, ^19^ This paper focuses on the former, with the aim of understanding and exploring women's experiences, in order to inform service developments and future provision, where TOP has until recently been provided exclusively in a hospital setting.

## Method

A qualitative research design offered flexibility to women in discussing their experiences of TOP in their own words.^20^ Recruiting sites – one community sexual and reproductive health (SRH) clinic, two hospital‐based clinics in one NHS area – served an urban/peri‐urban population, with women typically travelling for <1 hour to attend, and had been offering home self‐management for approximately 18 months prior to this study. Specialist health professionals provided study details to women attending for medical TOP ≤63 days, under Ground C of the 1967 Abortion Act (i.e. for psychosocial rather than medical indications). Over a 6‐month recruitment period (January–July 2014), 135 women completed an ‘opt‐in’ contact form, agreeing to be contacted by C.P. at a later date. C.P. made contact approximately 2 weeks later to arrange an interview. Six women were ineligible, two explicitly withdrew, and 81 were un‐contactable within the specified 6‐week timeframe. We did not conduct purposive sampling beyond the aim of recruiting an equal number of women who had presented in each of two clinical contexts (hospital and community SRH), comparison of which was of interest to the broader study. Interviews were conducted 3–5 weeks following TOP, in a location of the woman's choosing or by telephone. Recruitment and interviewing continued until data saturation was achieved; that is, when no new findings emerged from new data collected. Written consent was obtained prior to the interviews.

A flexible topic guide was developed in light of literature reviews and input from the study Advisory Group. Interviews explored TOP experiences, including interaction with health professionals, home self‐management and contraception.^17^ Interviews were digitally recorded and transcribed in full. Participants received a £15 voucher as compensation for their time.

A thematic analytical approach informed by the Framework method^21^ was undertaken by C.P. and J.H., who repeatedly read interview transcripts, before meeting to discuss and compare interpretations, and identify key themes. A coding framework was developed and applied to transcripts based on initial themes identified. Coded data sets were further analysed to identify linkages between themes and explore similarities and differences across accounts. Nvivo 10 qualitative data analysis software (QSR International 2012, Melbourne, Vic., Australia) facilitated coding and data management. Where data extracts are presented, identifiers in brackets indicate participant identifier and age.

## Results

We conducted in‐depth interviews with 44 women who had recently undergone medical TOP ≤63 days,*** and who had self‐managed passing the pregnancy at home. Key characteristics of participants are outlined in Table 1.

**Table 1:** Sample characteristics

Age (years)	Total (*n* = 44)	(% of total)
Mean	26.8	
Median	23	
Range	18–43	
**Highest education attained**		
Secondary school	14	31.9%
Further education (e.g. college)	13	29.5%
Higher education (i.e. university)	13	29.5%
Postgraduate	4	9.1%
**Relationship status (at interview)**		
Single	8	18.1%
Cohabiting/married	18	40.9%
In relationship (not cohabiting)	17	38.7%
Separated	1	2.3%
**Employment status**		
In paid employment	31	70.5%
Student	7	15.9%
Not in paid employment/full‐time parent	6	13.6%
Children	15	34.1%
Previous termination of pregnancy	7	15.9%

Issues relating to self‐management were clustered around two broad themes: women's introduction to self‐management at the clinic, and experiences of self‐management at home. Our analysis addresses different components of each, including: misoprostol self‐administration at clinic; choosing home self‐management; expectation‐setting by health professionals; getting home; self‐management and monitoring treatment progress; support for self‐management; and home pregnancy testing to confirm completion.

### Introducing self‐management at the clinic

#### Misoprostol self‐administration: ‘I was happy to do it myself’

For most participants, a degree of self‐management began with vaginal self‐administration of misoprostol at clinic. There was variation in the degree to which women felt this was optional and/or standard practice. Most saw it as relatively unproblematic and were relieved to self‐administer. However, some described feeling nervous about inserting the tablets correctly and preferred the nurse to do this:

I says ‘well, to be honest, I would rather *you* did, because at least I know it's done properly’. So she went, ‘that's no problem’. (SR21, 43)

Self‐administration, combined with a relatively brief appointment, led some to question why misoprostol could not be provided to take home at the mifepristone appointment, rather than having to re‐attend 1–2 days later:

Why does it have to take that long if I'm putting the pills in myself? […] Why can they not give you that on that day and say ‘tomorrow, stick these four pills up you? ‘Just to go in and do that for five minutes seems pointless to me. (H111, 26)

#### Why home self‐management? ‘I'd rather be in the privacy of my own home’

Some participants reported initial surprise that home self‐management was an option. The vast majority of women said that the privacy and comfort of their own surroundings was their preferred option:

What they'd said was – and I was in agreement – ‘You'd probably be way more comfortable at home. […] You *can* do it in a hospital environment…’ But 100% I'd much rather be at home with a telly and some ice‐cream. It's good to have the option, but definitely at home [you're] much more comfortable in your own bed.(SR22, 21)

Some participants were concerned with making undue use of NHS resources.

I didn't want to go to the hospital because I didn't want to take up a bed for six hours. I was really conscious of wasting the NHS's time, because I felt… like that was not necessary, and it was my own fault. (SR20, 21)

Some women also preferred to go home because they felt uncomfortable about the hospital TOP service's location adjacent to maternity services, and were worried about being judged by service users.

There was, again, variation in the extent to which women perceived home self‐management as optional. Some understood this to be the default treatment mode, with day wards reserved for exceptional cases. This included a minority who felt that they had not been given, or been able to request, day ward treatment.

I really wanted to stay in, but I never got the option for it. I think they maybe should let people stay in to definitely make sure it's all away […] I never mentioned it because I knew they weren't going to let me stay in. They were like ‘it's very rare for people to stay in’. So they were just like ‘see you later, go home’ basically. (H115, 22)

However, the presentation of home self‐management as the default care pathway left most feeling relatively safe and unconcerned:

I don't remember feeling like I had a choice. It pretty much just sounded like: ‘this is the normal procedure, this is what we do’. So I wasn't concerned. (H107, 30)

#### Expectation‐setting: ‘I felt fully informed’

For all women who self‐managed at home, information and advice from health professionals were crucial in assuring their comfort with this option. Participants described how health professionals had provided substantial amounts of information, with the appointment feeling like ‘lots of bits of paper, lots of pills’ (SR20, 21). As well as providing analgesics, much of the information related to expectation‐setting and advising women of circumstances in which they should seek help, which left most participants feeling appropriately informed:

They explain everything to you. I got a brown bag *full* of information. She spoke to me for about 20 minutes: emergency numbers, absolutely everything. (H104, 23)

As a result of health professionals’ frank descriptions, participants also reported having a clear sense of what was ‘normal’:

She was very matter‐of‐fact about it, she said: ‘Within 15 minutes once you'd inserted the tablets you'll start getting symptoms and the pain will build up. You'll probably start bleeding quite a lot. Then, over the next four hours, you should pass a very large clot, or several large clots. Once that's happened the pain should start dissipating […] They give you a scenario where you're bleeding out more than you should be and therefore you should be phoning up. (SR23, 38)

Where experiences at home were congruent with what women had been given to expect, they reported these experiences as broadly acceptable. After they had received these descriptions, factors about which participants said that they had continued to feel anxious or unsure included what expulsion would feel like, and what the pregnancy tissue would look like (see below).

### Home self‐management

Women's accounts of self‐management focused around pain, bleeding and passing the pregnancy, echoing experiences from clinic settings.^22^ Here we focus on how these experiences were shaped by their passing the pregnancy at home.

#### Getting home: ‘I started feeling quite ill in the car’

Participants described a range of experiences when travelling home from the clinic following misoprostol administration. Several felt nauseous or started cramping, while others recalled severe pain beginning:

Oh it started in the car […] I was in agony, I was saying to my partner ‘oh my god, it's kicking in, I'm getting really bad pains!’ So I got home and I just got into my pyjamas and went straight into bed (H206, 25)

While some women were relatively untroubled by the onset of symptoms during the journey – viewing it as a necessary evil, endured in order to get home – others felt frightened, upset or exposed. For women who knew no‐one with a car and/or could not afford a taxi, coping with the onset of pain and bleeding on public transport was particularly challenging and frustrating:

When I was on the bus I could feel that I was bleeding a lot […] I was really tired. I was just kind of, y'know: ‘I've spent 45 minutes on a bus for a two‐minute appointment..?’ (SR06, 27)

#### Being at home: ‘Being at home is really the best thing’

The vast majority of women said they valued the option to return home, as it had afforded them dignity and privacy that they expected would be absent in hospital:

It's such a physical and emotional process so, y'know, at home's better. *Hugely*. You basically need to like make friends with your toilet for eight hours, so it kind of works at home better. (SR02, 37)

[It was] *definitely* the right decision. I think that would be so much more traumatic if you did that in a hospital. *(C.P.: In what sort of way, d'you think?)* I just think it would be so… not ‘humiliating’, that's not the right word, but just so public. I was such a *mess*. I would be so embarrassed for people to see me like that… ‘(SR01, 21)

While not an absolute, those who reported that they might have preferred hospital‐based care were also those whose experiences were less congruent with the expectations set by clinic staff, and who did not necessarily feel that they had been given a choice.

#### Monitoring treatment progress: ‘Like two almighty periods in eight hours’

Many women noted the importance of the information provided by nurses which prepared them for the process, and enabled them to assess its progression:

It's good to do it at home, but I think a good level of honesty from the nurse [is helpful], because it's on the *cusp* of being cope‐able with. (SR23, 38)

In the absence of a nurse's assessment, bleeding was perceived as an indicator of efficacy. Having been briefed that bleeding was likely to be very heavy, some women had concerns about whether treatment had worked when bleeding was relatively light. A minority described surprise at the volume of bleeding, and some difficulty managing this:

That day was absolutely horrific. I bled so much. I didn't think it'd be as much as that and as much clotting and stuff. I was literally sitting on the toilet for half an hour at a time, because every time you stood up it just was pouring out […] You couldn't get to the bathroom fast enough, it was just coming in force. […] I had to go in the shower cause I'd leaked, and then you're standing in the shower and it's just pouring out and obviously you're seeing all the clots and I'm like ‘oh my god!’ (H202, 22)

Several participants reported that – as clinic staff advised they would – they had recognised when they had passed the bulk of the pregnancy tissue, and this had brought relief. Others were unsure whether this had occurred, and whether the TOP had been successful. Some women reported unease at the unfamiliar sensation of passing larger clots:

Y'know, sometimes if you've got your period, and you're sitting still for ages, and you stand up and you can kind of feel it? It was sort of like that. But I was on the toilet and it almost felt a little bit like… jelly, a little bit clotted. (SR10, 26)

Many women reported being advised by health professionals not to look at what they had passed, or that there would be nothing to see at such an early gestation. A small minority reported seeing what they believed to be a recognisable fetus, were distressed by this, and regretted looking:

[I] just felt compelled, that I had to look. So that's when I knew [TOP had been effective]. […] In hindsight I wish I hadn't looked but I did, and that was probably the most traumatic thing I've ever seen or done. I thought ‘what on earth..?’ (H201, 35)

#### Support in person: ‘going through it together’

Participants reported that they had all been strongly advised to have an adult with them for 24 hours after taking misoprostol, and the majority were supported at home to varying degrees by their partner (and a smaller number by a female friend/family member). Some reported that male partners had found the process challenging – feeling ‘useless’ or ‘helpless’ (H107, 30) – and some voiced a preference for hospital care, even where this was not the woman's preference. Those in relatively new relationships felt embarrassment that their partner had seen them go through the more visceral aspects of medical TOP, but nevertheless felt grateful that they had been able to go through it together at home.

Although most women had someone stay with them, some had preferred to be alone, and felt that having another person present would create additional distress:

[Friend] wanted to stay but I told her to go home, and I was so glad I did, because if she'd been in here and seen me screaming like that, she'd have phoned an ambulance. *She* would've panicked. (H201, 35)

Women in this position nonetheless had someone nearby and available by phone.

#### Support at a distance: ‘Knowing that I could pick up the phone at any time helped’

Many participants reported being informed at clinic that telephone support would be available from clinic staff if needed, though not all had required it:

[The] senior nurse gave me her mobile number and told me I could ring her if I wanted, if I had any questions, or if I was worried about anything, or if anything unexpected happened […] So I thought that was a really nice, like, ‘OK, I'm not alone here’. (SR01, 21)

For those who sought support, the reassurance provided by nurses was generally felt to be invaluable, particularly where experiences differed from what had been expected.

I phoned them […] and I says to them ‘listen, is this [ongoing cramping] normal? I know that you gave me painkillers but I expected to maybe take them on the [day] but not so many days later.’ And they were like ‘it's totally normal […] it's absolutely fine’. So again, [this] put my mind at ease. (SR21, 43)

Whether or not women had sought phone support, they reported feeling reassured that this was an option, and that they would not be required to explain their situation, as those answering the call would be aware that they were undergoing TOP.

#### Home pregnancy testing: ‘Thankfully it came back negative, so it's all done and dusted’

All participants had been provided with a low‐sensitivity urine pregnancy (LSUP) test to be performed 2 weeks after TOP. Some reported initial anxieties around self‐administering the test:

At first I did think ‘what if I do it wrong?’ and ‘what if I still am?’ and ‘would I prefer the professionals to do it?’ But you couldn't really do it wrong. And the symptoms sort of went away anyway, so I knew. So that was fine. (SRH21, 43)

Several women had not taken the test as they felt the physical signs of pregnancy had dissipated, and thus judged that the treatment had been successful. Most said they were happy with the self‐test, because it both confirmed that they were no longer pregnant and meant they would not require further contact with TOP services.

## Discussion

### Main findings

There is an urgent need to better understand what the shift toward self‐management in TOP care means for women. Our analysis demonstrates that women see home self‐management as beneficial, offering a potentially more comfortable space in which to go undergo TOP, and the opportunity to have support from a partner, friend or family member. Our findings highlight potential areas for improvement, primarily around the degree to which misoprostol self‐administration and home self‐management are conveyed to women as optional. Our analysis suggests that, although home was the preferred option for most, it is essential that the alternative of hospital care is made clear. This potentially requires a delicate balancing act for health professionals between normalising home self‐management and facilitating joint decision‐making and choice.

### Strengths and limitations

A limitation to the study is that all women were recruited from one NHS health board area, where a significant proportion of women self‐manage TOP ≤63 days and, at the time of fieldwork, home self‐management had been an option for over a year. It also drew on a relatively small sample, which did not allow for comparison with hospital‐managed care. Given the body of literature that suggests a tendency for recipients of healthcare to evaluate any care they receive positively,^23^ doing so may have produced a quite different picture. However, the richness of the data generated has enabled identification of a range of experiences, and new areas as yet unexplored in the literature on first trimester medical TOP.

### Interpretation (in light of other evidence)

Our findings add weight to existing evidence which has suggested that women undergoing TOP ≤63 days are largely amenable to self‐management and that, although they may experience some initial anxieties, they tend to express overall satisfaction.^2^, ^24^, ^25^, ^26^ Crucially, our analysis suggests that women value being given choices and feeling involved in decisions about their treatment,^4^, ^14^, ^17^, ^27^, ^28^ and goes some way to address concerns raised by providers about how women might cope at home.^18^ Our analysis also highlights that women's perceptions of their overall experiences were more negative when self‐management was not their preferred option, or when they did not feel involved in treatment decision‐making. This brings out the significance of offering women options in the TOP pathway, including whether and how to self‐administer misoprostol (i.e. vaginally or sublingually/buccally), and where to pass the pregnancy.

Our findings are supported by existing literature which has highlighted the vital role health professionals play in information provision and expectation‐setting.^27^, ^28^, ^29^ The results also underscore the importance to women embarking on TOP of feeling that they know what to expect, are in control, and that they have and are having an experience congruent with what they perceive health professionals have led them to expect. Our findings resonate with research which suggests that much of women's anxieties around TOP are grounded not in the decision to end the pregnancy but in apprehension concerning an unknown process.^27^, ^28^, ^29^, ^30^ Clearly informing women of what to expect of TOP is crucial to ensuring their safety and comfort, and to facilitating that sense of control.^2^, ^27^, ^28^ However, health professionals also need to convey the potential for *variation* in experiences of medical TOP, and are therefore required to strike a delicate balance between the specific detail that (some) women want and the range of experiences that they may face. Effectively striking this balance simultaneously affords women greater agency in the process, and maintains the place of first trimester TOP self‐management within the bounds of medical care.^19^

One current gap in this regard appears to be around experiences of passing larger clots/pieces of pregnancy tissue. It may be that this remains a relatively taboo aspect of the process, which health professionals find more challenging to verbalise; or it may be that this has not previously been flagged to providers as information which women undertaking self‐management want. Either way, this emerged as a significant, and for many women surprising, component of first trimester medical TOP.

Our analysis suggests that the comfort and privacy afforded by self‐management enabled women to cope with treatment in a way that suited them, without concerns about how they were being perceived by strangers, as they might in a hospital setting. This echoes the suggestion that self‐management can facilitate alternative experiences of TOP.^2^ Significantly, our findings shed further light on the essential role of available support (in this case by telephone) from health professionals during and following TOP.^2^ Given the potential variability of women's experiences of pain and bleeding, it is vital that those undergoing self‐management have the option to seek advice and reassurance as needed. The invaluable role that this support played for women in our study also underlines the implications for those to whom such a service is not available, and where TOP is not legally provided.^4^

Safe medical regimens offer advantages to women undergoing first trimester TOP, and the potential to develop self‐management further. In the British context, however, the current interpretation of the 1967 Abortion Act requires TOP medications to be administered on NHS premises. Hence, the opportunity to extend self‐management – to include, for example, home administration of misoprostol – is currently constrained, despite the significant issues an additional clinic visit can create for women.^31^ Given the significant changes in provision in the 50 years since it was passed, we echo the suggestion^14^ that the 1967 Abortion Act and its interpretation should be revisited, in order to facilitate further improvement in patient‐centred TOP care.

## Conclusion

This study explored the experiences of women who had self‐managed medical TOP ≤63 days of gestation, in a context where provision has until recently been hospital‐based. Our findings highlight that women valued self‐management, from self‐administration of misoprostol at clinic, through passing the pregnancy, to confirming TOP completion via a self‐performed LSUP test. It also brings to the fore the need to maintain high standards of information provision and appropriate support for women undergoing TOP in this model.

### Disclosure of interests

None declared. Completed disclosure of interests form available to view online as supporting information.

### Contribution to authorship

The paper was devised by C.P., and the first draft written by C.P. in consultation with J.H. S.C., J.L. and A.G. provided comments on this draft, and all authors approved the final version.

### Details of ethics approval

The study was reviewed and granted ethical approval by the Centre for Population Health Sciences Ethics Committee, University of Edinburgh (3 March 2014).

### Funding

The study was funded by the Chief Scientist Office for Scotland, grant number CZH/4/906. The research was conducted while C.P. was a Research Fellow at the University of Edinburgh, and the paper drafted while she was a Research Associate at the MRC/CSO Social and Public Health Sciences Unit, University of Glasgow (MC_UU_12017/11, SPHSU11).

## Notes

1 We deliberately use ‘Britain’/’British’ here, rather than ‘UK’, as the latter includes Northern Ireland, where access is much more severely restricted.

2 In some circumstances a woman may remain in hospital as a ‘day case’: for example, if she has a higher likelihood of medical complications, if she lives more than an hour from a hospital, if there are concerns about her safety or she has no‐one to be with her, if she speaks limited English.

3 Our sample thus represents approximately 5% of all women (*n* = 867) who had medical abortion ≤63 days in the recruiting area within the 6‐month recruitment period (figure provided by Information Service Division, personal communication 8 February 2017). Two interviewees were excluded from the analysis presented here as they had received hospital treatment.
